# Therapeutic Horseback Riding Crossover Effects of Attachment Behaviors with Family Pets in a Sample of Children with Autism Spectrum Disorder

**DOI:** 10.3390/ijerph14030256

**Published:** 2017-03-03

**Authors:** Jessie D. Petty, Zhaoxing Pan, Briar Dechant, Robin L. Gabriels

**Affiliations:** 1 Children’s Hospital Colorado, 13123 E. 16th Ave, Aurora, CO 80045, USA; jessie.petty@childrenscolorado.org (J.D.P.); Zhaoxing.pan@childrenscolorado.org (Z.P.); briar.dechant@childrenscolorado.org (B.D.); 2 Department of Child & Adolescent Psychiatry, University of Colorado Anschutz Medical Campus, 13001 E. 17th Place, Aurora, CO 80045, USA

**Keywords:** autism spectrum disorders, therapeutic horseback riding, pet relationships, human-animal interactions, animal assisted interventions

## Abstract

The unique needs of individuals with autism spectrum disorder (ASD) have implications for animal welfare. This nested pilot study examined the effects of a randomized trial of 10-week therapeutic horseback riding (THR) intervention versus a no-horse barn activity (BA) control group on children’s behaviors with family pets. Sixty-seven (THR *n* = 31; BA *n* = 36) participants with ASD (ages 6–16 years) with one or more family pet, were enrolled from a larger trial (*n* = 116) following their randomization to intervention groups, stratified by nonverbal intellectual ability. A consistent caregiver completed questionnaires about participants’ interactions with their household pets pre- and post-intervention. Caregivers of THR group participants reported significant improvements in participants’ caring actions with the family pet compared with the BA group (*p* = 0.013; effect size = 0.74). Engaging with horses during a standard THR intervention protocol may generalize to improving caring actions toward family pets in children and adolescents with ASD.

## 1. Introduction

There can be a strong bond between a child and their pet, as can an animal provide comfort and a sense of security that in turn can facilitate positive social and stress management [1]. However, children with Autism Spectrum Disorder (ASD) have specific impairments in communication and social skills, along with the presence of restricted interests, insistence on sameness, and repetitive behaviors that can affect their social interactions with others, including family pets [2]. Specifically, ASD diagnostic impairments involve difficulty in knowing how to relate to others in pro-social ways and understanding how their behaviors impact others [3]. This population also has a tendency to be over- or under-responsive to environmental stimulation and have difficulty generalizing skills learned in one setting to apply in another [4,5,6]. The nature of these unique diagnostic characteristics can jeopardize interactions between children with ASD and a pet living in the family home, which has been reported as a caregiver concern in this population [7,8]. Further, this population has been reported to “be rough with animals and treat them like inanimate objects”, if they do not have specific interventions to teach appropriate care of animals [9].

There is emerging empirical evidence that animal-specific interventions (AAI) can have beneficial effects on the unique challenges of the ASD population [3,10]. O’Haire reviewed 14 empirical AAI studies with populations affected by ASD that included dogs, guinea pigs, llamas, rabbits, and horses as the intervention animal [3]. While each AAI study was distinctly analyzed, commonalities existed across study results, showing increases in social interaction and communication skills as well as decreases in ASD symptom severity, stress, and problem behaviors [3]. A more recent review of 28 additional AAI studies with the ASD population by O’Haire revealed that the most common intervention animal was a horse (55% of studies reviewed) and that the most common outcome was an improvement in social interaction (79% of 22 studies) [10].

The present study reports additional results from the largest randomized trial (*n* = 127) to date of Therapeutic Horseback Riding (THR) with the ASD population (ages 6 to 16 years) [11]. That paper reported results demonstrating the efficacy of the THR intervention compared to an active control on decreasing irritability and hyperactivity behaviors as well as improving social cognition, social communication, and number of words and new words spoken during a language sample [11].

The purpose of the current study was to determine whether 10 weeks of a THR group intervention compared to an active control intervention (i.e., barn activity (BA) group with no horse contact) could crossover to positive changes in participants’ interactions with their family pet(s). It was hypothesized that participants in the THR intervention would demonstrate an increase in the number of positive interactions with their household pets compared to participants in the control group.

## 2. Materials and Methods

### 2.1. Participants

A subset of participants (*n* = 67) from a more extensive randomized trial study were included in this institutional review board-approved pilot project [11]. Participants were included if caregivers identified that there was at least one family pet living in the home. All participants were 6–16 years of age and met study criteria for autistic or Asperger’s disorder as described in the main study [11]. Informed consent was obtained from each caregiver and participant. A total of 31 participants were included from the THR group and 36 participants from the BA control group. As part of the larger study, participants were randomly assigned to either the THR or a BA control group, based on their nonverbal intelligence score, and BA group participants were informed that they would receive two free riding lessons at the end of the study [11]. Participants were not separately randomized for this pilot study of pet attachment. Similar to the large gender discrepancy in the general population of those affected by ASD, the majority of this sample consisted of males. Participant demographics are reported in Table 1.

### 2.2. Pet Attachment Measure

Information regarding attitudes toward and care for animals associated with the child–pet interactions taking place in the household was gathered using the caregiver report “Child’s Attitude and Behavior toward Animals” (CABTA) [12]. The CABTA part C (animal cruelty items) is the only section that has published test–retest reliability and comparison validity data [12]. Test–retest reliability of part C items and its factors showed high reliability (*p* < 0.001; r^2^ = 0.98) and good comparison validity with the Parent’s Account of Children's Relationships with Animals (PACRA) in its ability to detect animal cruelty. However, there is no known reliability or validity data for part B items of the CABTA. The CABTA consists of a 21-item likert-type rating scale, asking caregivers to assess their child’s behaviors related to animals [12]. This instrument also includes questions about the child’s history of acting cruelly towards animals [12]. A modified version of this measure was used for the purposes of these analyses, which omitted four questions of the CABTA. Specifically, the following two questions (i.e., “My child has ridden a horse” and “My child likes fishing”) were not included because they related to exposure to animals, not the interaction taking place [12]. Also, the following two questions (i.e., “My child has harmed (a) small insects (b) other non-domestic animals (c) other people’s pets (d) his/her pet(s)” and “My child has harmed animals”) were not included due to their historical nature [12]. A total of 14 questions were analyzed for the purposes of this study. Each question on the CABTA measure was coded 0, 1, 2, 3, and 4, representing not applicable, rarely, sometimes, often, and always [12]. Two summary scores were calculated and analyzed based on this questionnaire: Animal attachment score (AATS) and animal abuse score (AABS). AATS is the sum of questions 7, 8, 9, 11, 12, and 13, while AABS concerns items 15, 16, 17, 18, 20, 22, 23, and 24. Two items (i.e., questions 13 and 22) had to be inversely coded before calculating the summary scores for each respective category. The maximum possible score for the AATS was 24 and 32 for AABS. A higher AATS score and a lower AABS score indicate more optimal functioning in the areas measured.

### 2.3. Study Design

Caregivers were asked to complete the CABTA as a part of the larger study along with a battery of other study assessments within one month pre- and post-study sessions [11]. The 10-week study interventions were as follows: (1) The THR group consisted of a one-hour lesson that included learning topics related to horses (e.g., horse emotions) and horsemanship skills while riding horses for 45 min in small group settings of three to four participants, followed by a 15 min horse care activity (e.g., grooming horses and helping their volunteer equine handlers to put away tack). This 15-min routine involved leading their horse out of the arena to an assigned station where they secured the lead rope to a post, removed the tack from their horse, and used grooming tools to care for their horse. In the tack room, each horse had a locker marked with the horse’s name and picture to designate for participants where materials were to be put away. (2) The BA group also consisted of a one-hour lesson at the same riding center in a small group setting with volunteers for each participant in which they adhered to the same lesson as the THR group each week (i.e., horse emotions). Similar to the THR group, the BA group included activities focused on learning about horses, safe rules for being around horses, and horse care, but with no horse contact [11]. For example, one week’s curriculum helped BA group participants learn about the tools and practices for properly grooming their horse and then they were given an opportunity to “groom” a life-sized toy horse. See Gabriels et al. [11] for additional details about the intervention protocols.

### 2.4. Power of the Study

There was no a priori sample size determination for this nested study. Post hoc power analysis indicates that 31 THR and 36 BA control participants provided 80% power at 5% significance to detect between-group differences if the Cohen’s effect size is 0.7 or greater using two sample *t*-test.

### 2.5. Statistical Analysis

Data are presented as mean (standard deviation (SD)) for continuous measures or percentages for categorical responses. Imbalance between the two groups was examined using two sample student *t*-tests or a chi square test as appropriate. The intent-to-treat analysis was deemed as the primary analysis and the completer analysis as the sensitivity analysis. A linear mixed effects model with unstructured covariance was used for analysis of primary outcomes, where the fixed effects consisted of outcome evaluation time (i.e., baseline or end of study), and study group (THR or BA) as classification variables, and their interaction term. Test of the time by group interaction term (i.e., whether one group has greater post-intervention change in outcome as compared to another group) assessed the efficacy.

## 3. Results

There were no differences in the number of pets reported being in the home and the majority of pets in each group were dogs. There were no significant differences between the two groups with respect to participants’ nonverbal intelligence-quotient (IQ), co-existing psychiatric diagnoses, number of pets, or type of pet owned.

There was significant difference at baseline in AATS (mean 13.59 for THR vs. 15.78 for BA, *p* = 0.02). The AATS score of participants in the THR group significantly improved from 13.59 to 15.4 after intervention (*p* = 0.003) while the score of BA participants showed no trend of improvement (*p* = 0.69). Table 2 shows that the between-groups difference in the post-THR change is statistically significant (Effect Size (ES) = 0.74, *p* = 0.013), indicating that THR has a favorable effect on AATS scores. Analysis of each question revealed significant between-groups difference in post-intervention change for two items of AATS scores relating to the child having a good relationship with the pet and the child acting in a caring manner toward their pet—questions 8 (*p* = 0.008) and 9 (*p* = 0.01). At baseline, participants in the THR group had a lower mean score (2.59 (1.05)) for question 8, which was significantly different (*p* = 0.02) from 3.17 (0.91) in the BA group. Participants in the THR group significantly changed from baseline to after intervention for either question 8 (*p* = 0.005) or 9 (*p* = 0.02) while there was no significant change in the BA group (*p* = 0.38 and 0.19 respectively for questions 8 and 9). There were no statistically significant between-group differences in baseline and post-intervention change in AABS. The completer analysis (*n* = 21 participants for THR, *n* = 24 participants for BA) produced the same results as the Intent To Treat (ITT) analysis.

## 4. Discussion

This was hypothesized based on previous study findings of increased social behaviors with others resulting from AAI in the ASD population [3,10]; findings that children and adolescents with ASD who participated in a 10-week THR intervention would generalize appropriate social interaction skills with horses, thus exhibiting an increase in their positive interactions with their family pets compared to those who participated in a 10-week similar intervention with no horse interaction. In this study, THR participants showed significantly more improvements acting in a caring manner toward household pets as reported by caregivers. This is the first known study to examine the crossover effects of THR in children with ASD on their caring behaviors toward domestic pets.

This study is limited by the nested design, which resulted in the small sample size and imbalance of baseline AABS total score between THR and BA. The estimate of efficacy of THR could be biased to overestimation due to a ceiling effect. This is particularly true for the analysis of question 8, asking if the child had a good relationship with their pet, for which the BA group mean score was close to the ceiling of four. Also, it is unclear how using a modified version of the CABTA measure may have affected its overall validity. The CABTA was designed to evaluate the maltreatment of animals, rather than caring toward animals. There are a limited number of measures in current circulation for the topic of pet attachment. Given this, future studies may want to consider using the Companion Animal Bonding Scale (CABS) [13]. The CABS, a validated measure that includes items directly related to the area significance, was demonstrated in these analyses. For example, questions target themes of positive relationships with animals as well as caring for a pet [13]. Although the CABS is intended to be a self-report measure, it could easily be modified to allow for caregiver reporting as needed for this population.

Moving forward, measuring how caregivers and family functioning are also impacted by the human–animal interaction may be useful. Emerging research presents data suggesting that while animal interaction may be significantly helpful for children with ASD, it may also serve to benefit others in the home in terms of family relationships [14].

## 5. Conclusions

Study results support previous research findings, showing an increase in positive social interactions as a result of an AAI on individuals with ASD [3,10]. The AAI field would benefit from additional studies exploring if social interaction improvements resulting from an AAI can crossover to other settings in the ASD population.

## Figures and Tables

**Table 1:** Participant demographics of all enrolled participants.

Characteristic	THR	BA	Total	*p*-Value ^a^
Participants, *n*	31	36	67	
Age, y, mean (SD)	10.95 (3.42)	10.01 (2.66)	10.45 (3.05)	0.21
Gender, *n*, M/F	27/4	33/3	60/7	0.54
IQ, mean (SD)	86.45 (24.56)	89.03 (19.54)	87.84 (21.87)	0.63
Community psychiatric diagnoses				
Autism	26	26	52	0.37
Asperger’s	5	10	15	0.25
Latino/Hispanic	4	9	13	0.21
Race				0.49
American Indian or Alaska Native	0	1	1	
Asian/Hawaiian/Pacific Islander	0	2	2	
Black or African American	1	0	1	
White	27	29	56	
Multiracial	0	1	1	
Other	1	2	3	
Missing Data	2	1	3	
Type of pet				0.62
Dog	19	19	38	
Cat	7	7	14	
Bird	2	0	2	
Fish	6	3	9	
Other	3	3	6	

Note: BA = barn activity; F = female; IQ = intelligence-quotient; M = male; THR = therapeutic horseback riding; SD = standard deviation. ^a^ Two-tailed *p*-value from two sample *t*-test, chi-squared test, or Fisher exact test, as appropriate.

**Table 2:** Effect of THR on animal care score and animal abuse propensity scores ^a^.

Intent-To-Treat Analysis ^a,b^	THR			BA (Control)			Interaction (Efficacy)		
	Baseline	EoT	Change	Baseline	EoT	Change			
	Mean (SD)	Mean (SD)	Mean (SD) ^d^	Mean (SD)	Mean (SD)	Mean (SEM) ^d^	Mean (SEM)	*p*-Value	ES ^c^
Animal abuse propensity score (sum of C15, C16, C17, C19, C20)	5.52 (3.40)	5.48 (4.03)	0.28 (0.59)	4.53 (3.42)	4.63 (2.75)	0.26 (0.56)	0.02 (0.81)	0.984	0.01
Animal care score (Sum of B7, B8, B9, B11, B12 and (4-B13)	13.59 (4.36)	15.40 (3.51)	1.84 (0.58) **	15.78 (3.17)	15.17 (4.20)	−0.22 (0.55)	2.06 (0.80)	0.013	0.74
B8. My child has a good relationship with our pet/s	2.59 (1.05)	3.08 (0.91)	0.48 (0.16) **	3.17 (0.91)	2.96 (0.91)	−0.13 (0.15)	0.62 (0.22)	0.008	0.76
B9. My child acts in a caring manner towards our pet/s	2.81 (0.96)	3.20 (0.91)	0.35 (0.15) *	3.17 (0.85)	2.88 (1.12)	−0.19 (0.14)	0.54 (0.21)	0.012	0.76

^a^ Sample means and standard deviation were reported for baseline and end of treatment (EoT). Mean and standard errors of change and the time by group interaction are from mixed effects model analysis of baseline and EoT data for all the outcome variables. ^b^ Analyses included all participants who were randomized into the THR or BA group of the primary trial, eligible for this nested study and had either baseline and/or EoT assessment (31 THR and 36 BA participants were analyzed; 27 THR and 36 BA participants had baseline data and 25 THR and 24 BA participants had EoT data). ^c^ Effect size (ES) is calculated $\left(2\times t\;value\right)/\sqrt{DF}$ (DF: degree of freedom) from the contrast of the time by group interaction. ^d^ *p < 0.05, **p < 0.01.

## References

[1] Walsh F. (2009). Human-Animal Bonds II: The Role of Pets in Family Systems and Family. Fam. Process.

[2] American Psychiatric Association (2013). Diagnostic and Statistical Manual of Mental Disorders: DSM-5.

[3] O’Haire M.E. (2013). Animal-assisted intervention for autism spectrum disorder: A systematic literature review. J. Autism Dev. Disord..

[4] Gal E., Cermak S.A., Ben-Sasson A., Gabriels R.L., Hill D.E. (2007). Sensory processing disorders in children with autism. Growing Up with Autism.

[5] Radley K.C., O’Handley R.D., Ness E.J., Ford W.B., Battaglia A.A., McHugh M.B., McLemore C.E. (2014). Promoting social skill use and generalization in children with autism spectrum disorder. Res. Autism Spectr. Disord..

[6] Carnahan C.R., Hume K., Clarke L., Borders C. (2009). Using Structured Work Systems to Promote Independence and Engagement for Students with Autism Spectrum Disorders. Except Child.

[7] Carlisle G.K. (2015). The Social Skills and Attachment to Dogs of Children with Autism Spectrum Disorder. J. Autism Dev. Disord..

[8] Carlisle G.K. (2014). Pet Dog Ownership Decisions for Parents of Children with Autism Spectrum Disorder. J. Pediatr. Nurs..

[9] Grandin T., Fine A.H., O’Haire M.E., Carlisle G., Bowers C.M., Fine A. (2015). The roles of animals for individuals with autism spectrum disorder. Handbook on Animal-Assisted Therapy.

[10] O’Haire M.E. (2017). Research on Animal-assisted intervention for autism spectrum disorder, 2012–2015. Appl Dev. Sci..

[11] Gabriels R.L., Pan Z., Dechant B., Agnew J.A., Brim N., Mesibov G. (2015). Randomized controlled trial of therapeutic horseback riding in children and adolescents with autism spectrum disorder. J. Am. Acad. Child Adolesc. Psychiatry.

[12] Guymer E.C., Mellor D., Luk E.S.L., Pearse V. (2001). The Development of a Screening Questionnaire for Childhood Cruelty to Animals. J. Child Psychol. Psychiatry.

[13] Anderson D.C. (2007). Assessing the Human-Animal Bond: A Compendium of Actual Measures, New Directions in the Human-Animal Bond Series.

[14] Wright H., Hall S., Hames A., Hardiman J., Mills R., Mills D., PAWS Project Team (2015). Pet Dogs Improve Family Functioning and Reduce Anxiety in Children with Autism Spectrum Disorder. Anthrozoös.

